# Supplementary material for: Multi-sensor information fusion localization of rare-earth suspended permanent magnet maglev trains based on adaptive Kalman algorithm
Source: PLoS One. 2023 Nov 28;18(11):e0292269. doi: 10.1371/journal.pone.0292269 (PMC10684099; doi:10.1371/journal.pone.0292269)
Supplement: Attachment — Submitted filename: Comments.docx [file pone.0292269.s002.docx]

1.The references in 2023 are not available, and the latest references are added.

2. Magnet maglev train speed is fast, how to process information quickly, how to calculate the realization.

3. The font of axis in Figure 4-15 is too small to read clearly.

4.The estimation of covariance Q and covariance R by adaptive Kalman filter is relatively mature. In this paper, only the estimation of Q is not convincing enough.

5. In Figure 7, no processor is seen. What processing method is adopted after data collection? Please specify.

6. In the review, the application of Kalman, the development of the suspension vehicle and the application of multiple sensors are described. The application of Kalman in the maglev vehicle is missing, isn't it?

7. ME and EMSE are used in this paper for evaluation. Can you specify the actual error data, the actual position and the position gap using this method?
